# YM155 potently triggers cell death in breast cancer cells through an autophagy-NF-kB network

**DOI:** 10.18632/oncotarget.3638

**Published:** 2015-05-06

**Authors:** Eloïse Véquaud, Céline Séveno, Delphine Loussouarn, Lucie Engelhart, Mario Campone, Philippe Juin, Sophie Barillé-Nion

**Affiliations:** ^1^ CRCNA, UMR INSERM U892 / CNRS 6299 / Université de Nantes, Team 8 « Cell survival and tumor escape in breast cancers », Institut de Recherche en Santé de l'Université de Nantes, Nantes, France; ^2^ Service d'Anatomie Pathologique, HGRL, CHU, Nantes University, Nantes, France; ^3^ Institut de Cancérologie de Nantes, Centre de lutte contre le Cancer René Gauducheau, Boulevard Jacques Monod, Nantes, France

**Keywords:** breast cancer, therapy, *ex vivo* assay

## Abstract

Specific overexpression in cancer cells and evidence of oncogenic functions make Survivin an attractive target in cancer tharapy. The small molecule compound YM155 has been described as the first “Survivin suppressant” but molecular mechanisms involved in its biological activity and its clinical potential remain obscure. We herein show that YM155 exerts single agent toxicity on primary breast cancer cells grown in an *ex vivo* assay preserving tumor microenvironment. *In vitro* assays indicate that YM155 more efficiently triggers cell death in breast cancer cells (including these with stem-cell like properties) than in non tumorigenic mammary cells. YM155-induced cell death is critically dependent on autophagy and NF-kB but independent of p53 and it coïncides with DNA damage an a DNA damage response in p53-proficient cells. Our results point out a crosstalk between NF-KB and autophagy controlling YM155-induced death in breast cancer cells and argue for the potential use of YM155 as a genotoxic agent in breast cancer therapy.

## INTRODUCTION

Survivin gene, i.e BIRC5, expression is upregulated in many human tumors and this correlates with metastatic spread, tumor invasiness, and poor prognosis associated with treatment resistance. While its role in restricting the execution of cell death has not been fully resolved, it is clear that Survivin participates in cell cycle control especially during mitotic spindle checkpoint and cytokinesis. In addition, its barely detectable levels in normal adult tissues makes Survivin an attractive target for pharmacological intervention in cancer therapy [1, 2].

YM155 was the first drug reported to block Survivin expression [3]. This small imidazolium compound was initially identified from a phamacological screen based on BIRC5 promoter inhibition and described as a first in class “Survivin suppressant”. YM155 has been demonstrated to exert antitumor activity, to suppress Survivin expression and to induce tumor cell apoptosis, in various human cancer models. It has already completed phase 2 clinical trials for various kinds of cancers which validates its safety [reviewed in 2]. These revealed a modest anticancer activity as a single agent and trials in combination with paclitaxel and carboplatin in solid tumors are now ongoing. It is thus of importance to evaluate YM155 activity against specific types of cancers and to define more accurately how it may exert its effect on cancer cells. This is apposite as recent studies suggest that suppressing Survivin expression was not the main target of YM155 in cancer cells. In addition, the exact modes of cell death induced by YM155 remain essentially uncharacterized.

Tight regulation of both NF-κB pathway and autophagy process is necessary for maintenance of cellular homeostasis. In cancer cells, deregulation of both pathways is frequently observed and is associated with tumorigenesis and tumor cell resistance to cancer therapies [4, 5]. Importantly, both are induced under cellular stress and ensure homeostatic responses in controlling each other through positive or negative feedback loops. Autophagy, that is a self-degradative process recycling cytoplasmic components through autophagosomes formation and their fusion with lysosomes, generally acts as a energy sensor and protects cell integrity but when unfavorable conditions persist, it may act as a cell death pathway. Its role in cancer is dual from tumor-suppressive activity in early oncogenesis to contribution to drug resistance in advanced cancer [6, 7, 8]. NF-kB pathway also interplays in cancer cells' survival control and its activation constitutes a rapidly inducible first line of defence against cellular stress and have important role in resistance to cancer therapies [5, 9]. Of note, apoptosis is a main cell death program triggered by chemotherapy treatments [10] and many molecular links between this biochemically well-defined executive process and autophagy or NF-kB pathway have been reported. Apoptosis involves the Bcl-2 family proteins as major regulators of the mitochondrial apoptotic pathway and/or the TNF-R family in the extrinsic apoptotic pathway. It relies on a proteolytic caspase-dependent cascade, to demantle cells and endow them with specific morphological characteristics [11, 12]. Importantly, under stressfull conditions such as treatment using chemotherapy, an intricate interplay between the homeostatic pathways NF-kB and autophagy and the apoptotic executive process may take place in cancer cells that will ultimately dictate their fate between cell death or survival. Identifying how innovative anticancer agents exert their effect at cellular level is of major importance in anticipating their efficacy in cancer or relevant synergistic combinations.

In this study, we have evaluated the antitumor activity of the small-molecule Survivin suppressant, YM155 in a relevant preclinical model of breast cancers and explored the signalling pathways on which its activity relied. We provide the first evidence that YM155 triggered cell death in primary breast cancer cells embedded in their environnement in a majority of human breast tumors. Interestingly, we also report that among mammary cells, the transformed and the cancer-initiating cells are preferentially targeted by YM155. Consistent with previous reports, we further observe that the autophagy process delineates YM155-induced cell death in a p53-independent way despite DNA damage occurence, but we also demonstrate that the canonical NF-kB pathway potently controls YM155-induced cell death, upstream the autophagy process. Overall, our results point out for the first time that YM155 induces cell death in primary human breast cancer cells and that a NF-KB and autophagy network controls its activity.

## RESULTS

### Preclinical evaluation of YM155 treatment on human mammary tumors in an *ex vivo* organotypic culture assay

**Figure 1:**
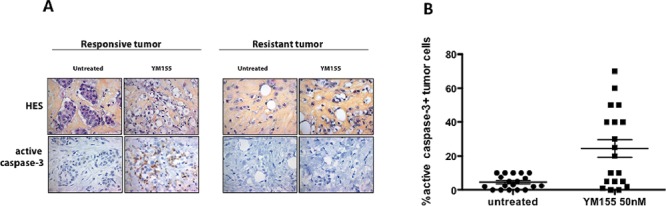
YM155 induces cell death in primary breast cancer cells using an organotypic 3D assay *Ex vivo* cultures of primary human breast tumors (*n* = 19) were cultured 48 h with 50 nM YM155 or not treated (untreated). Samples were then analyzed for cancer cell morphology by Hematoxylin-Eosin-Saffron staining (upper panel) and active caspase-3 positivity by immunohistochemistry lower panel), as shown in **A.** for a YM155-responsive (left panel) or a resistant tumor (right panel). **B.** Data including 19 tumors are represented as % of carcinomatous cells positive in each specimen, in both untreated and YM155-treated conditions.

We recently developed *ex vivo* organotypic cultures of human primary breast tumors in which integrity of tumors embedded in their microenvironment, is properly preserved [15] and we began this study by examining the response of 19 breast tumors to YM155 using this assay in order to evaluate its clinical potential. Briefly, fresh tumors were rapidly cut in thin slices and incubated in full medium alone or with 50 nM YM155 for 48 h. Tumor slices were then paraffin-embedded and analyzed for morphologic integrity by HES staining and active caspase-3 expression, as a marker of cell death response (apoptosis more particularly), by standard immunohistochemistry methods. 19 consecutive primary tumors from patients with untreated breast cancer were included of which 17 were diagnosed as positive for estrogen receptorα (ERα). IHC scores of positive cells for cleaved caspase-3 and the standard hematoxylin-eosin-saffron staining were determined in the epithelial tumor cells compartments. Such results obtained for two different individual tumors, one responsive and one resistant to YM155 treatment, are presented in Figure 1A. Among the cohort of 19 tumors, active caspase-3 staining displayed a mean score of 24, 4% (ranging from 0 to 70%) in YM155-treated tumors compared to 4, 5% (ranging from 0 to 10%) in untreated (vehicle-treated) tumors (Figure 1B), indicating that YM155 significantly induced apoptosis in primary breast tumor cells. We fixed a positive threshold above 20% of cleaved caspase 3 positive tumor cells, which is superior to the highest score in untreated samples. Under these conditions, YM155 treatment identified 3 subgroups of breast tumors: the first group exhibiting a response to YM155 above 20% in cleaved caspase-3 staining, defined as sensitive tumors, included 10 tumors and the second including 7 tumors with caspase-3 scores under 5% was considered as YM155-resistant tumors. For two tumors, the caspase-3 score was determined as 10%, corresponding to an intermediate response. Importantly, a robust correlation was noted between the percentage of active caspase-3 tumor cells and tumor cell integrity, as evaluated with HES staining performed on the same sample (*p* = 0.0003). This strongly suggests that the active caspase-3 was a relevant marker of YM155-induced cytotoxicity on the tumor samples in this *ex vivo* test.

10/19 tumors (52, 6%) were thus significantly sensitive to YM155 used at 50 nM. Among them, 8 were ERα-positive and 2 ERα-negative and all resistant or intermediate tumors tested were ERα-positive. This clearly indicated that YM155 was a potent cell death inducer in primary breast cancer cells in presence of their microenvironment in more than half tumors included. This also revealed that 7/19 (36, 8%) tumors were apparently resistant to YM155-induced cell death and highlighted the need to unravel the signalling pathways underpinning cancer cells' response to YM155.

### YM155 treatment preferentially triggered cell death in breast cancer cells through an autophagy-dependent pathway

**Figure 2:**
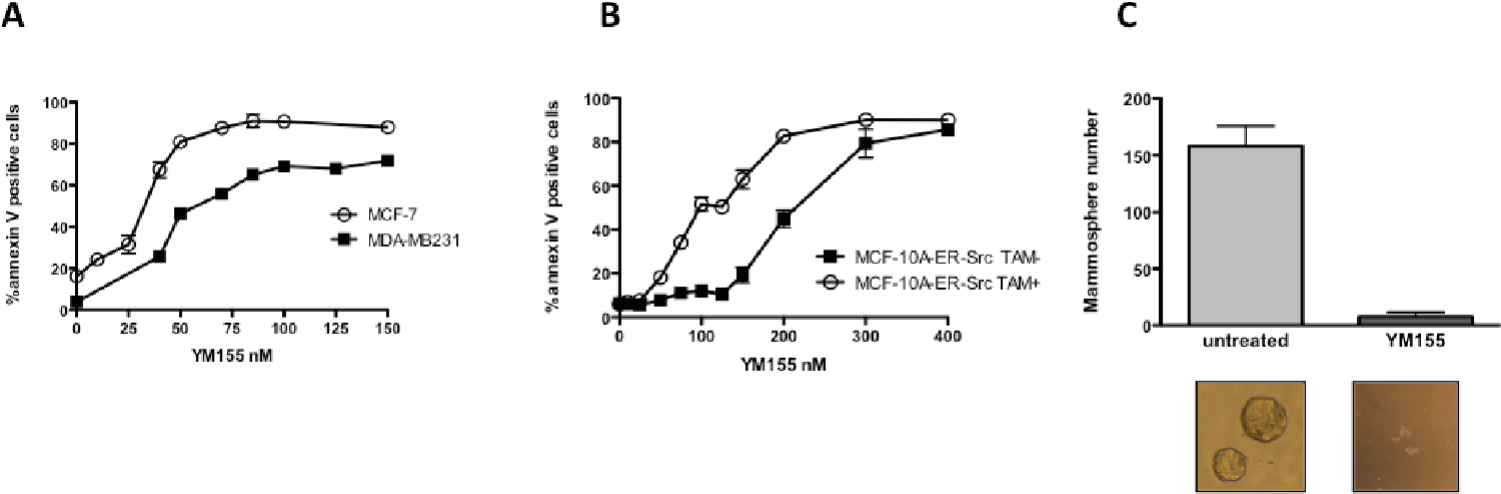
YM155 preferentially induces cell death in breast cancer cells and targets cancer cell with stem-like phenotype MCF-7, MDA-MB231 **A.** and MCF-10A-ER-Src treated or not by 40H-TAM **B.** were cultured for 48 h in presence of increasing YM-155 concentrations. Annexin-V/IP assay was then performed to quantify dead cells in each condition. **C.** Mammosphere formation was analyzed by microscopy for the number of mammospheres formed upon YM155 treatment at 50 nM for 4 days using MCF-7 cell line in comparison with untreated cells. Data are the results of 3 independent experiments. Mammosphere images obtained in both conditions are shown in the lower insert.

To further investigate how YM155 triggers cell death, we used human breast cancer cell lines. MDA-MB231, Cal51 and MCF-7 breast cancer cell lines were first tested for their sensitivity to YM-155 when grown in a 2D *in vitro* assay. Using cell death assay and flow cytometry analysis, we observed that a strong cell death response was achieved in cells treated for 48 h at 50 nM. Dose-response analysis indicated that YM155 concentration equal to 40 nM for MCF-7, 50 nM for MDA-MB231 and 70 nM for Cal-51, was sufficient in killing 50% of corresponding breast cancer cells (Figure 2A and data not shown). Of note, decreased expression of Survivin and MCL1 proteins was detected in YM155-treated using these YM155 concentrations (Figure 4A and Supplementary Figure S1 respectively).

In contrast the non-tumorigenic mammary cell line MCF-10A (MCF-10A-ER-Src cultured in the absence of TAM) was less sensitive and a concentration of 200 nM was needed to kill half cells (Figure 2B). Interestingly, transformation of these cells by induction of the expression of v-src oncogene by TAM treatment, as previously described [13], led to an increase in their sensitivity to YM155, from 200 nM in the parental cell line to 100 nM as concentrations needed to kill 50% of cells (Figure 2B). This indicates that transformed cells were more sensitive than their non-transformed counterpart. Moreover, as breast cancer cell lines may harbor a subpopulation of cancer initiating cells with features resembling these of stem cells, we assessed whether YM155 could target this subpopulation in the MCF-7 cell line using an *in vitro* mammosphere formation assay. Importantly, we observed a dramatic decrease in mammosphere formation after YM155 treatment at 40 nM compared to control cells (Figure 2C). This suggests that transformed cells and among them those presenting stemness features might be more sensitive and that they more critically rely on YM155's targets for their survival than their untransformed-counterpart

**Figure 3:**
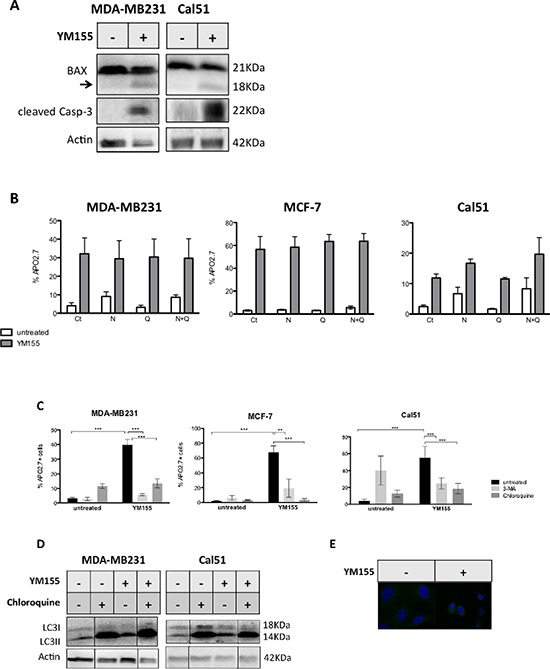
YM155 triggers an autophagy-dependent cell death pathways in breast cancer cells **A.** MDA-MB321 or Cal51 cells were treated by YM115 at 40 nM and 70 nM respectively for 48 h and cleavage of caspase-3 and Bax proteins were evaluated by immunoblot analysis. The arrow on Bax immunoblot indicates a cleaved form of Bax with 18KDa MW. **B.** MDA-MB321, MCF-7 and Cal51 cells were first incubated with 10 μM of the pan-caspase inhibitor QVD-Oph (Q) and/or with 1 μM of the RIP1 inhibitor Necrostatin-1 (N) for 3 h then treated with respectively 50, 40 or 70 nM of YM155 for 48 h in presence of the above inhibitors (YM155) or not (untreated). Cell death assays were then performed and % of dead cells determined in each indicated condition. No significant inhibition of cell death was observed (*n* = 3). **C.** A similar pretreatment with the autophagy inhibitors Chloroquine (25 μM) or 3-MA was applied to MDA-MB231, Cal51 or MCF-7 cells, followed by a 48 h-treatment by YM155 at above concentrations (YM155) or not (Un), before cell death evaluation. **D.** Similar culture conditions were applied before performing immunoblot analysis for LC3 evaluation. When indicated, Chloroquine pretreatment (25 μM) was realized 3 h before adding YM155. **E.** LC3 was evaluated by IHC in YM155-treated (+) at 40 nM for 48 h versus untreated (−) Cal51 cells.

We further sought to define which cell death pathways YM155 treatment could activate in breast cancer cells. We first observed that cleaved caspase-3, that is a hallmark of apoptosis, appeared in YM155-treated cells consistently with results obtained in *ex vivo* cultures (Figure 3A). In addition, the proapoptotic Bcl-2 family protein BAX, was also cleaved upon YM155 treatment (Figures 3A et 5D). These results argue for the activation of caspase cascade triggered by YM155 treatment. However, we could not observe any significant protection of cancer cells from cell death using the pan-caspase inhibitor Q-VD-OPh (Figure 3B). Same results were obtained using Z-VAD-FMK as another pan-caspase inhibitor (data not shown). We thus evaluated whether necroptosis or autophagy contibue to cell death onset as these processes are functional in presence of caspases inhibition. Using necrostatin-1 as a specific inhibitor of RIP1, the main kinase involved in necroptosis pathway, in combination with caspase inhibitor or not did not lead to significant cell protection against YM155 treatment. These results rule out necroptosis as the apical activated cell death pathway (Figure 3B). In contrast, blocking autophagy process using either 3-MA or chloroquine, strongly protected cancer cells from YM155-induced cell death in the 3 cell lines (Figure 3C). Pretreatment using Bafilomycin A1 could also protect cancer cells from YM155-induced cell death (data not shown). We thus evaluated the autophagic flux in YM155-treated cells compared to untreated cells using LC3 marker (its cytoplasmic-associated form LC3I and its autophagosome-associated form LC3II) by immunoblot analysis. Decreased expression of LC3-I and increased expression of LC3-II upon YM155 treatment suggested that YM155 increased the autophagic flux in breast cancer cells (Figures 3D and 5D). Moreover, IHC analysis revealed LC3 expression as cytoplasmic vesicles in YM155-treated cells compared to untreated control cells, that is a hallmark of autophagy activation (Figure 3E). In addition, strong LC3-II accumulation was obtained upon chloroquine treatment a due to efficient autophagy blockade that was maintained when combined with YM155. Finally, we also detected accumulation of the autophagy adaptator p62/SQSTM1 upon YM155 treatment for 48 h (Figure 5D). Altogether, these results indicate that YM155 activates an autophagy-dependent cell death pathway that harbors caspase activation but that does not strictly rely on caspase activity for cell death execution.

### YM155 treatment had genotoxic effects and triggered DNA damage response in breast cancer cells

**Figure 4:**
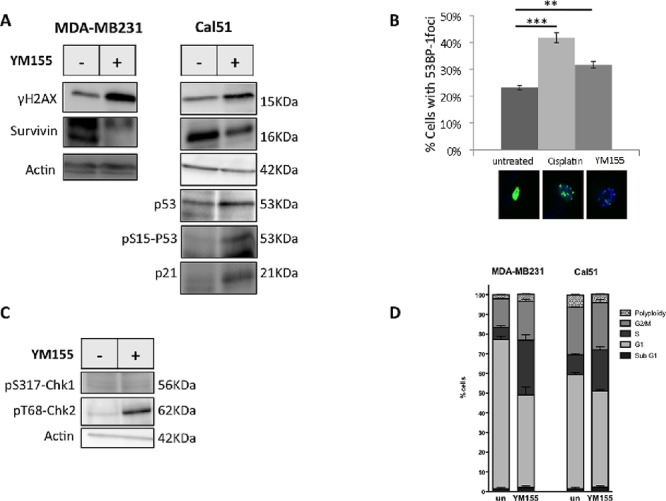
YM155 induces DNA damage and initiates DNA repair response **A.** S139-phosphorylated-H2AX and Survivin proteins expression were performed by immunoblot analysis in YM155 treated-cells using the p53-mutated MDA-MB231 cell line and the non mutated-p53 cell line Cal51. P53, its S15 phosphorylated form, and its gene target p21 were also assessed in these latter p53 proficient cells. **B.** Initiation of DNA repair was evaluated by counting 53BP-1 nuclear foci formed in GFP-53BP-1-expressing Cal51 after indicated treatment. Cisplatin (50 μM) was used as positive control. Representative images of flourescent cells in each conditions are also inserted in the figure. **C.** Phosphorylation of Chk1 and Chk2 on S317 and T68 respectively, upon YM155 treatment was evaluated by immunoblot analysis in comparison with untreated Cal51 cells. **D.** Cell cycle analysis was performed in cell lines using IP staining and flow cytometry analysis after 48 h incubation with YM155 compared to untreated control cells.

**Figure 5:**
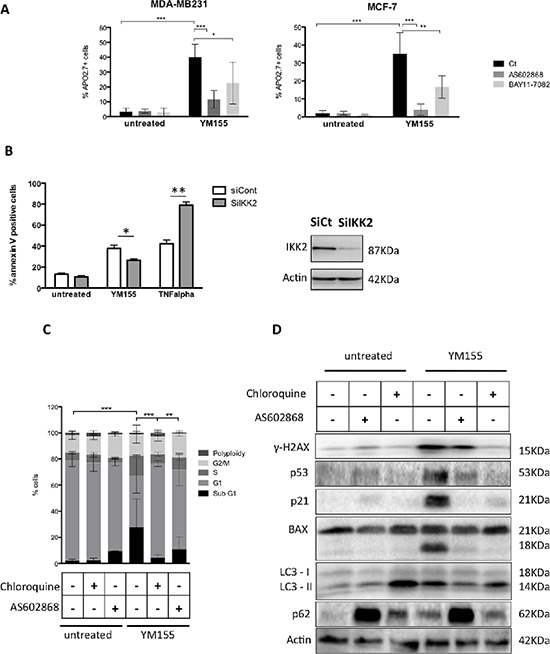
IKK2 contributes to YM155-induced cell death **A.** Cell death assays were performed in MDA-MB231 or MCF-7 cells pretreated with the IKK2 inhibitors AS602868 (10 μM) or BAY11-7085 (5 μM) for 3 h before exposed, for additional 48 h, to 50 and 40 nM YM155, respectively or not (untreated). **B.** YM155-induced cell death was evaluated in MCF-7 cells after IKK2 knock-down by RNA interference (left panel). TNF-alpha (50 ng/ml) was used as a positive control. IKK2 protein depletion was verified by immunoblot analysis (right panel). (C and D) Cell cycle **C.** and immunoblot **D.** analysis were performed in MCF-7 cells pretreated by AS602868 (AS) or Chloroquine before exposure to YM155 (40 nM) for 48 h as previously described.

As YM155 was recently suspected to target DNA [16] we evaluated whether YM155 had genotoxic effect on breast cancer cells and might trigger DNA damage response. We indeed observed the phosphorylation on S139 of histone H2AX (γH2AX) upon YM155 treatment using immunoblot analysis (Figure 4A) as well as nuclear γH2AX-foci formation by IHC (data not shown). Importantly, this happened in both mutated or wild-type p53 cell lines. In addition, eventhough both Survivin and MCL1 protein levels (Figure 4A and Supplementary Figure S1A) were decreased upon YM155 treatment, the pancaspase inhibitor QVD-Oph did not protect cells from DNA damage (data not shown). We then sought for a DNA damage response upon YM155 treatment. Using Cal51 cells transfected by a plasmid coding for the protein fusion GFP-53BP1, we detected significant 53BP1 nuclear foci formation upon YM155 treatment (Figure 4B) compared to untreated cells and cisplatin-treated cells used as positive control. Moreover T68-phosphorylated Chk2 was also detected after YM155 treatment. In contrast no S317-phosphorylated Chk1 could be detected (Figure 4C). These results argue for the recruitment of early markers of DNA breaks and initiation of DNA repair mainly involving ATM-Chk2 pathway. Using the wild-type (wt)-p53 cell line Cal-51 or MCF-7, we evidenced p53 accumulation and S15 phosphorylation upon YM155 treatment (Figure 4A, right panel and Figure 5D, respectively). Importantly increased expression of p21 appeared in these cells after YM155 treatment. We thus sought whether YM155 might impact cell cycle progress. Consistently, as shown in Figures 4D and 5C, YM155 treatment significantly slowed down cell cycle progress with cells preferentially accumulating in S-phase. Of note, this also occurred in p53 mutated cells. Altogether these data indicate that YM155 induced caspase-independent DNA damage in replicating cells and triggered DNA damage response in breast cancer cells leading to cell death independently of p53 status.

### Blocking NF-kB pathway strongly protected cancer cells from YM155-induced cell death

As NF-kB pathway stands at the cross-road of various signalling pathways including cell death and autophagy in cancer cells, we decided to decipher whether it could contribute in breast cancer cells' response to YM155. Unexpectedly, using the IKK2 inhibitor AS602868, we observed that pretreating cells with this inhibitor before adding YM155, strongly protected cells from YM155-induced cell death (Figure 5A). The same effect was obtained using the pan-IKK inhibitor BAY11-7085. We checked that AS602868 effectively targeted canonical NF-kB pathway in breast cancer cells by evaluating its capacity to counteract TNFα-induced activation of NF-kB transcription using a NF-kB gene reporter assay and to potentiate TNFα-induced cell death. Both assays were conclusive, arguing for the specificity of AS602868 to potently inhibit conventional NF-kB pathway (Supplementary Figure S1B and C). Importantly and consistent with these results, RNA interference targeting IKK2 (Figure 5B right panel) also led to significant decrease of YM155-induced cell death (Figure 5B, left panel). In the opposite, cell death induced by TNFalpha was increased upon IIK2 depletion, arguing for efficient inhibition of NF-kB pathway. In an attempt to unravel the impact of NF-kB pathway in autophagy-dependent YM155-induced cell death, we compared chloroquine and AS602868 effects on cancer cells response to YM155. We first observed that AS602868 as well as chloroquine pretreatment significantly impaired YM155-dependent cell cycle blockade as shown for MCF-7 in Figure 5C and in MDA-MB231 (data not shown). In addition, these inhibitors could partially protect cancer cells from DNA damage triggered by YM155 treatment and both of them modulated the subsequent p53 response in these cells. Indeed in MCF-7 cells, p53 accumulation was decreased upon pretreatment with AS602868 or chloroquine before YM155 exposure (Figure 5D). Consistent with this effect, YM155-induced p21 protein accumulation was also impaired in cells pretreated with AS602868 or chloroquine. Moreover, consistent with results obtained in cell death assays, these treatments also prevented Bax cleavage. Finally, AS602868 treatment limited YM155-induced LC3II accumulation, suggesting that NF-kB pathway and IKK2, may cross-talk with autophagy signalling upon YM155 treatment (Figure 5D). Blocking NF-kB pathway unexpectedly lead to strong p62 accumulation in untreated cells, showing that NF-kB and autophagy pathways can potently talk to each other. Overall, our results suggest that NF-KB or autophagy blockade spoiled p53 response in breast cancer cells upon YM155 treatment and determined cancer cell fate through interconnected signalling pathways.

## DISCUSSION

Survivin overexpression in many human tumors and its correlation with advanced disease, treatment resistance, and poor outcome, have highlighted its potential value as a relevant target for cancer therapy. In this line, some natural or chemical Survivin suppressors have been identified [2, 17]. Among them, the small molecule YM155 came out of a high-throughput screening for inhibitors of *survivin* promoter activity and phase I clinical trials using this compound, conducted in heavily pretreated cancer patients provided evidence that an objective tumor response could be achieved in some cases with an overall favorable toxicity profile. Phase II studies of YM155 monotherapy recently reported more or less disappointing results depending on the origine of tumors and on the stage of the disease [18, 19] and clinical studies based on combination including YM155 and conventional chemotherapy are now ongoing. Importantly, in contrast to initial statement, it appeared that Survivin was not the only target of YM155 in cancer cells. As such, MCL1 was identified as the pivotal YM155 target in myeloma cells [20]. This raises the questions of whether this molecule could be relevant in cancer therapy and how it may exert its potential anticancer activity.

In this report, we focused on defining YM155 activity in breast cancer cells. For the first time, we describe YM155 effect on human primary breast cancers in an *ex vivo* model and we provide evidence that YM155-induced cell death results from a complex signalling network involving autophagy process and NF-KB pathway in response to YM155-induced DNA damage. Importantly, this effect appeared independently from p53 status of cancer cells.

We first observed that YM155 induced significant cancer cell death in more than half (10/19) human primary breast tumors in an *ex vivo* organotypic culture assay that maintains tumor integrity including breast cancer cells and their microenvironement [15]. Indeed, using IHC analysis, we found a strong correlation between morphological changes in carcinomatous cells and cleaved caspase-3 positive staining suggesting that cell death was a major process of YM155 sensitivity. Interestingly, cleaved caspase-3 that is a hallmark of apoptosis, was also detected in response to doxorubicin in a similar *ex vivo* model, indicating that cell death pathways with caspase activation are major response of tumors to anticancer treatment [21]. In addition, in breast cancer cell lines upon YM155 treatment, we observe not only caspase-3 activation, as previously reported [22], but also Bax cleavage, suggesting that activation of a cascade of proteases including calpains involved in Bax proteolysis [23, 24] and caspases, happened in response to YM155. However, caspase inhibition using pharmacological inhibitors could not protect cancer cells from cell death, excluding apoptosis as the main executive cell death pathway triggered by YM155 treatment. The RIP1 inhibitor Necrostatin-1 was also inefficient in blocking YM155-induced cell death, even in combination with a pan-caspase inhibitor, demonstrating that necroptosis was neither involved in YM155-induced cell death pathway. In contrast, blocking autophagy process either upstream with 3-MA or downstream with chloroquine, potently counteracted cell death onset due to YM155 treatment. These results are consistent with those of Wang or Cheng and colleagues who in addition, report an autophagy-dependent activation of caspases induced by YM155 [25, 26]. Collectively these results and ours provide evidence that autophagy process controls cell death triggering upon YM155 treatment. As frequently observed with chemotherapy, YM155 induces autophagy activation in breast cancer cells. In many cases, autophagy constitutes a strategy to adapt to and cope with stress. However, since autophagy is a lysosomal degradative pathway, that may lead to lysosome membrane disruption (LMP), it can be an alternative mechanism of programmed cell death that precedes apoptotic cell death [27]. LMP results in the release of hydrolases in the cytoplasm leading to protein digestion and activation of additional enzymes including caspases and to cell lethality [28]. Chloroquine that in relation with its lysosomotropic property inhibits hydrolase activity, probably blocks the proteolytic cascade triggered by YM155 treatment and by this way, cell death onset. Interestingly, profound changes in lysosomal compartment affect cancer cells and this may be the cause of their higher sensitivity to YM155 compared to non transformed cells we observed. Thus linking YM155 activity and lysosomes biology represents an appealing perspective that we are currently exploring.

Since YM155 may behave as a DNA intercalating agent, capable of interfering with DNA relaxing in an acellular assay [29], we further explored whether YM155 induced DNA damage in breast cancer cells. Our data clearly indicated that YM155 treatment triggered not only DNA damage but also initiated a DNA repair response. Of particular interest, when we turned to cell cycle analysis, we found that YM155 treatment induced an accumulation of replicating cells with DNA contents between 2n and 4n. This is consistent with previous demonstration of significant impact of YM155 on DNA synthesis [16]. Collectively, recent reports and our results strongly argue for classifing YM155 as a DNA damaging agent rather than as a pure Survivin suppressor. Importantly, our data indicate that YM155-induced cell death did not depend on p53 status of cancer cells and confirm the observation reported by Nakahara and colleagues [30].

Finally, using the NF-kB inhibitor targeting IKK2, we provide evidence that the canonical NF-kB pathway contributes to YM155-induced cell death. Indeed, targeting IKK2 by pharmacological inhibitors as well as RNA interference, strongly blunted YM155 cytotoxic effect. in stressfull conditions, IKK2 that belongs with IKK1 and NEMO to the IKK complex, is frequently activated leading to IkBα phosphorylation and the subsequent release of NF-kB dimeres whose nuclear translocation allows their transcriptional activity [31]. Interestingly, upon genotoxic stress, an ATM/ATR-dependent control of NEMO may drive the NF-kB response towards promotion rather than inhibition of cell death in triggering a complex NF-kB dependent transcriptional program that include both pro and anti-apoptotic genes [32]. In this line, NF-kB pathway can control TNFalpha release upon DNA damage then determining the final outcome of cancer cells [33]. Of major interest, our results indicate that both NF-kB or autophagy inhibition prevent breast cancer cells from cell cycle blockade and cell death triggering upon YM155 treatment. In addition, NF-kB inhibition in YM155 treated cells decreases autophagy activation. Our results argue for the control of YM155-induced cell death by NF-kB upstream the autophagic process and reveal the complex inteconnections between these signaling pathways. In addition, they indicate that eventhough YM155 triggers DNA damage, this effect may not be the initial event in the signaling cascade since inhibiting either NF-KB or autophagy pathways prevents DNA damage detection. Further experiments are needed to precisely unravel this signaling network that finally dictates cancer cell fate upon YM155 treatment but may also interfere with other genotoxic agents.

Altogether, our data provide evidence that YM155 is an effective cell death inducer in breast cancer cells, even in presence of their microenvironment and that both homeostatic pathways NF-kB and autophagy control the signaling pathway leading to cell death in YM155-treated cells. YM155, that is probably not a specific Survivin inhibitor, remains a valuable anticancer drug. Despite, a remarkable efficacy in preclinical studies, the first results of clinical trials based on YM155 used as single agent were disappointing. This possibly reflects the incompletely identified molecular targets of the drug and/or the absence of biomarkers for patient stratification. The functional evaluation of tumor response to YM155, in the way we report in this study, may help to identify patients that could benefit from this therapy. *Ex vivo* results will indeed help us to define the molecular determinants involved in the breast cancer resistance to first-line YM155 treatment. In addition, eventhough combining YM155 with either conventional anticancer drugs [19, 34, 35] or innovative molecules such as BH3 mimetics [36, 37] are already in progress in clinical trials with carboplatin and paclitaxel in patients with solid tumors, our results raise some concerns about relevant combination since NF-kB and autophagy pathways may interfere with those therapeutics.

## MATERIALS AND METHODS

### Reagents and cell lines

MCF-7 and MDA-MB231 cell lines were from American Type Culture Collection (ATCC, Rockville, USA), and Cal51 from DSMZ (Braunschweig, Germany). The ER-Src MCF-10A cell line was a generous gift of Dr Kevin Struhl (Harvard Medical School, Boston, USA). All cell lines were cultured following supplier's recommendations. The ER-Src MCF-10A cells contain an integrated fusion of the v-Src oncoprotein and the ligand-binding domain of estrogen receptor and are induced to rapidly transform when grown with 1 μM 4OH-TAM (Sigma-Aldrich, Saint-Quentin Yvelines, France), as previously described [13].

YM155, Necrostatin-1 and BAY11-7085 were purchased from Selleck Chemicals (Houston, USA) and the pan-caspase inhibitor Q-VD-OPh from R&DSystems (Abingdon, UK). Chloroquine, 3-Methyl-Adenin (3-MA), Bafilomycin A1 were purchased from Sigma-Aldrich (Saint-Quentin Yvelines, France). AS602868 was a generous gift of Merck-Serono International SA [14]. Antibodies against Survivin was purchased from R&DSystems (Lille, France), antibodies against LC3, p21, pS15-p53, pS317-Chk1, pT68-Chk2, IKK2, Actin, γH2AX from Merck Millipore (Saint-Quentin en Yvelines, France), antibodies against HSP90, p53 and cleaved (i.e. activated) caspase3 from Becton Dickinson (Pont de Claix, France), antibody against BAX from Dako (Courtaboeuf, France), and antibody against p62 from Santa Cruz (Heidelberg, Germany).

### Preclinical breast cancer *ex vivo* assay

Fresh human mammary samples were obtained from chemotherapy naive patients with invasive carcinoma after surgical resection at the Institut de Cancérologie de l'Ouest, René Gauducheau, Nantes, France. As required by the French Committee for the Protection of Human Subjects, informed consent was obtained from study patients to use their surgical specimens and clinicopathological data for research purposes, and the local ethic committee approved protocols. The tumors were cut into thin slices (250 μm) using a vibratome (Microm Microtech, Francheville, France) and incubated for 48 h with or without 50 nM YM155. Slices were then fixed in 10% buffered formalin and paraffin embedded. Sections (3 μm) were then cut for standard histological analysis assessed by hematoxylin-eosin-saffron (HES) coloration and immunohistochemistry analysis using cleaved caspase-3 antibody, as a cell death marker, as previously described [15]. Percentages of positive cells for cleaved caspase-3 staining among 200 carcinomatous cells were established. Expression for estrogen receptor alpha (ERα) was defined in parallel by IHC for diagnostic purpose.

### Cell death assays

Cell death was assessed either by Apo2.7 (Beckman Coulter, Grenoble, France) staining or Annexin-V binding assay (Miltenyi Biotec, Paris, France) performed according to manufacturer's instructions. Flow cytometry analysis was performed on a FACSCalibur using the CellQuestPro software in the CytoCell flow cytometry facility (SFR Bonamy, FED4203/Inserm UMS 016/CNRS 3556, Nantes, France).

### Cell cycle assay

Cells were treated by YM155 for 48 h (following pretreatment using either Chloroquine 25 μM or AS602868 10 μM, when indicated), then harvested and fixed overnight in PBS-FBS 12.5%-ethanol 50%. Ater washing in PBS, cells were resuspended in a solution of PBS-PI (Propidium Iodide 13.5 μg/ml). Flow-cytometry analysis was performed on a FACSCalibur by using the CellQuestPro software.

### Mammosphere formation assay

MCF-7 cells were grown in serum-free mammary epithelial cell growth medium containing DMEM-F12 (Sigma-Aldrich, Saint-Quentin, France) supplemented with B27 (Life Technologies, Saint-Aubin, France) and MEGM singlequots (Lonza, Verviers, France) for 7 days, as previously described (Campone, Mol Cancer 2010), in presence of YM155 (50 nM) or not. Mammosphere-forming unit were counted as number of mammospheres ≥ 50 μm. Data are mean ± sem of 3 independent experiments.

### Immunoblot analysis

Cells were resuspended in lysis buffer (1% SDS; 10mM EDTA; 50mM Tris-Hcl pH8, 1; 1mM PMSF; 10 μg.ml^−1^ aprotinine; 10 μg.mL^−1^ leupeptine; 10 μg.mL^−1^ pepstatine; 1mM Na3VO4 and 50mM NaF) and sonicated with Bioruptor apparatus from Diagenode. Protein concentration was measured using bicinchoninic acid (BCA protein assay, Pierce, Rockford, IL, USA). Fifty micrograms of proteins were loaded for each lane and separated by 10%, 12.5% or 15% SDS-PAGE, then electrotransfered to PVDF membranes. Western blot analysis was performed by standard techniques with ECL detection (Bio-Rad, Marne-la coquette, France).

### RNA interference

Cells were transfected using Lipofectamine RNAiMax™ 2000 (Life Technologies, Saint-Aubin, France) according to manufacturer's instructions. Medium was changed 6 hours later and compounds were added after 24 hours. The siRNA control and IKK2 were purchased from Thermo Fisher Scientific (St Leon Rot, Germany).

### Immunofluorescence imaging

Cells were plated onto glass coverslips and grown for 24 h prior to a 48 h treatment with YM155. After immunostaining using LC3 antibody and 488-Alexa goat-anti-mouse secondary antibody (Molecular Probes (Paisley, UK). 53BP1 expressing Cal51 cells were obtained after transient transfection using the GFP-53BP1c coding plasmid obtained from Dr Thomas Von Zglinicki (New Castle University, UK) (Nelson CC 2009). They were then plated onto glass coverslip, treated by the indicated agents for 48 h. In both cases, cells were counterstained with DAPI and images were viewed on a Zeiss Axiovert 200M microscope in the MicroPIcell imaging facility (SFR Bonamy, FED4203/Inserm UMS 016/CNRS 3556, Nantes, France). % of cells with GFP staining were established in 53BP1 expressing cells.

### Statistical analysis

Statistical analysis was performed using one-tailed paired Student's *t*-test and one-way ANOVA test on GraphPad Prism. Errors bars represent standard errors of mean (sem). The following symbols are used: *, **, *** that correspond to a *p* value inferior to 0.05, 0.01 or 0.001 respectively and ns for non statistically significant.

## SUPPLEMENTARY MATERIAL AND METHODS


