# Supplementary material for: YM155 potently triggers cell death in breast cancer cells through an autophagy-NF-kB network
Source: Oncotarget. 2015 May 6;6(15):13476–86. doi: 10.18632/oncotarget.3638 (PMC4537028; doi:10.18632/oncotarget.3638)
Supplement: Supplementary file 1 [file oncotarget-06-13476-s001.pdf]

SUPPLEMENTARY MATERIAL AND METHODS

Promoter-reporter activity assay

The ability of AS602868 to inhibit TNFalpha-induced NF-kB transcription activity was assessed using the luciferase based-NF-kB promoter assay from Panomics (eBioSciences, Paris, France), and the dual-luciferase reporter assay system from Promega (Charbonnières, France) as recommended by the manufacturers. Briefly, cells were transfected with the NF-kB promoter plasmid or its corresponding control, with a βGalactosidase coding plasmid using Lipofectamin 2000 (Life Technologies, Saint-Aubin, France), pre-treated by the AS602868 inhibitor followed by TNFalphaexposure for 48 h before measuring luciferase activity that was normalized by βGalactosidase activity.

SUPPLEMENTARY FIGURE

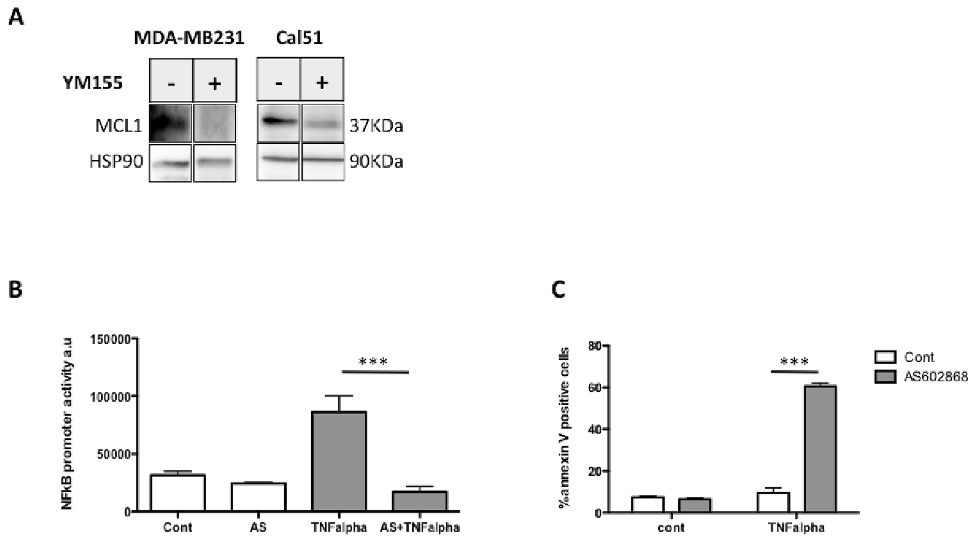

Supplementary Figure S1: AS602868 inhibits IKK2 in the NFkB pathway. A. MCL1 protein expression was performed by immunoblot analysis in YM155 treated-cells compared to untreated cells in MDA-MB231 and Cal51cell lines. **B.** MCF-7 cells were transfected with a plasmid coding for the NF-kB gene promoter, then pre-treated or not with for 3 h with 10 µM AS602868 (AS), before addition of 50 ng/ml TNFalpha for 48 h. **C.** Cell death assay was performed in MDA-MB231 cells pre-incubated for 3 h with 10 µM AS602868 or not, then exposed to TNFalpha (50 ng/ml) for 48 h or not (cont).
